# Dempster-Shafer Theory for the Prediction of Auxin-Response Elements (AuxREs) in Plant Genomes

**DOI:** 10.1155/2018/3837060

**Published:** 2018-11-01

**Authors:** Nesrine Sghaier, Rayda Ben Ayed, Riadh Ben Marzoug, Ahmed Rebai

**Affiliations:** ^1^Laboratory of Molecular and Cellular Screening Processes, Center of Biotechnology of Sfax, BP 1177, Sfax 3018, Tunisia; ^2^Faculty of Sciences of Gabes, University of Gabes, City Riadh Zerig, 6072 Gabes, Tunisia

## Abstract

Auxin is a major regulator of plant growth and development; its action involves transcriptional activation. The identification of Auxin-response element (AuxRE) is one of the most important issues to understand the Auxin regulation of gene expression. Over the past few years, a large number of motif identification tools have been developed. Despite these considerable efforts provided by computational biologists, building reliable models to predict regulatory elements has still been a difficult challenge. In this context, we propose in this work a data fusion approach for the prediction of AuxRE. Our method is based on the combined use of Dempster-Shafer evidence theory and fuzzy theory. To evaluate our model, we have scanning the DORNRÖSCHEN promoter by our model. All proven AuxRE present in the promoter has been detected. At the 0.9 threshold we have no false positive. The comparison of the results of our model and some previous motifs finding tools shows that our model can predict AuxRE more successfully than the other tools and produce less false positive. The comparison of the results before and after combination shows the importance of Dempster-Shafer combination in the decrease of false positive and to improve the reliability of prediction. For an overall evaluation we have chosen to present the performance of our approach in comparison with other methods. In fact, the results indicated that the data fusion method has the highest degree of sensitivity (Sn) and Positive Predictive Value (PPV).

## 1. Introduction

Plants are genetically very diverse group and are playing a vital role in nutrition and livelihood in particular for rural and tribal masses for employment and income generation In response to various developmental conditions and severe environmental changes by regulating gene expression. Transcription is at the core of physiological and developmental processes that requires well-coordinated players. Auxin is a major regulator of plant growth and development that plays important roles during all the stages of plant life and their action involves transcriptional activation. This phytohormone controls multiple fundamental aspects of the plant development [1] and environmental responses such as apical dominance [2], root development [3], phototropism, and gravitropism [4]. Also, Auxin is crucially involved in cell division, cell elongation, and cell differentiation [5]. The action of these plant hormone centres on the activation of early-response genes [6] and microarray studies has identified a large number of early Auxin-response genes [7]. Many players are implicated in the transcriptional mechanism in the regulation of Auxin target gene expression. Auxin-response element (AuxRE) is a key element which is necessary in this process. The first and second reactions involve recognition of this specific element which contains the core sequence TGTCTC [8].

The identification of AuxREs is one of the most important issues to understand the Auxin regulation of gene expression at the genome level. Cis-regulatory elements can be elucidated by experimental technologies in vitro such as ChIP-chip [9], ChIP-seq [10, 11], and ChIP-PET [12]. However, using laboratory techniques is laborious and the process requires significant time and resources [13]. This is why many computational methods have been developed to allow fast and efficient identification of hormone receptor regulatory elements [14, 15]. Computational prediction of TFBS motifs remains a central goal in bioinformatics and intensive efforts have been dedicated to identifying putative cis-regulatory elements.

Several algorithms have been developed for the detection of consensus sequences. They can be categorized into two main strategies [16, 17]: enumeration of short words (counting and comparing oligonucleotide frequencies) [18, 19] and probabilistic methods [20, 21]. Usually, motif finding tool identifies short DNA sequence ‘motifs' that are statistically overrepresented in regulatory regions (promoters) [21, 22]. A statistically overrepresented motif signify a motif that occurs more often than one would expect by chance [16]. Many computational approaches have been applied such as heuristic, greedy [23], and stochastic algorithms, some others used, expectation maximization (EM) [24], Gibbs Sampling algorithms [25], Hidden Markov model (HMM) [13], Bayesian network [26], Genetic algorithms (GA) [25], and others [16].

A pattern can be represented as a consensus sequence or a position weight matrix (PWM) [46]. PWMs are frequently applied for transcription factor binding site prediction [23, 47]. It describes the probability to find the nucleotides A,C,G,T on each position of a motif [48]. Searching pattern for matches with a PWM is more accurate than consensus string matching, but it also produces a large number of false positives [49, 50]. Other methods use localized distribution as a supplementary criterion to detect functional elements [51]. Over the past few years, a large number of motif identification tools have been developed, to name a few, MAPPER [52], AlignACE [21], MEME [53], Weeder [54], MotifSampler [55], and GAME [56]. Because of this diversity of algorithms and programs available, many studies present a comprehensive review of motifs predictors that provide comparison and guidance to researchers such as Stormo [48], Das and Dai [16], and tompa et al. [57]. These studies show that despite these considerable efforts provided by computational biologists, building reliable models to predict regulatory elements was always a challenge in task. Stormo and Zhao [57] suggested that the majority of the current approaches are not accurate or complete and it is necessary to find more accurate prediction methods with higher specificity and sensitivity. So a new bioinformatics framework is required. Tompa et al. [57] recommended the use of a few complementary tools and follow up the top motifs by combining information from different predictions. Hu et al. [58] discussed the limitations of motif discovery algorithms and developed a new one, named, EMD, which is more significant for shorter input sequences [59].

In this context, we propose in this work a data fusion approach for the prediction of Auxin-response elements. Our method is based on the combined use of Dempster-Shafer (DS) evidence theory and fuzzy sets. It consists of modelling detection uncertainty and fusing the features using DS combination rule.

## 2. Material and Methods

### 2.1. Training Set (Data Collection)

A training set of 64 experimentally verified that hormone response elements were collected from published data (Table 1). Whole genome dataset and upstream sequences of* Arabidopsis thaliana* were downloaded from TAIR (http://arabidopsis.org/).

Position weight matrix used for comparison tools was obtained from Ponomarenko and Ponomarenko [60]. Linear discriminant analysis was performed using SPSS (v. 16.0, Statistical Package for the Social Sciences, Chicago, IL, USA).

Microarray data of the primary response to Auxin in* Arabidopsis* was taken from Genevestigator database (https://genevestigator.com/gv/) [61]. Response in seedlings was selected: 1 *μ*M IAA for 1 h [62].

### 2.2. Implementation of the Algorithm

The main algorithm was implemented under the R environment language. All measurements were performed on a single CPU Intel Core i3 computer running at 2.8 GHz, with 6 GB main memory. The source code is available upon request.

### 2.3. Some Fundamentals of Dempster-Shafer Theory

The Dempster-Shafer (DS) evidence theory is a mathematical theory originated from the earlier works of Arthur P. Dempster in 1967 [63, 64] and extended by Glenn Shafer in 1976 [65]. DS theory can be considered as a generalization of Bayesian probability theory which uses the notions of imprecise, uncertain, and incomplete information. It has been applied in various domains such as medical diagnosis, image processing, and expert systems [66, 67]. DS theory can be used to combine information from different sources. DS theory uses ‘belief' rather than probability. ‘Belief' function is used to represent the uncertainty of the hypothesis. In DS theory, there is a finite set of N elements called the frame of discernment Θ = {H1, H2,…, H_N_}. It is a set of mutually exclusive and exhaustive propositions.

Information sources can distribute mass values on subsets of the frame of discernment. A numerical measure of uncertainty, termed basic probability masses, may be assigned to sets of hypotheses as well as individual hypotheses.

The mass functions verify the following constraints:

(1) 0≤mAi≤1m∅=0∑Ai∈2θmAi=1

where A_i_ designates a simple hypothesis Hi or composite hypotheses (union of simple hypotheses), A_i_ = 2^*θ*^.

If we consider two mass distributions m_1_ and m_2_ from two different information sources, m1 and m2 can be combined with Dempster's orthogonal rule, and a new distribution *m* = *m*_1_ ⊕ *m*_2_ is calculated in the following manner:

(2) $$\begin{array}{c} m\left(\;A_{i}\;\right)={\left(\;1-K\;\right)}^{-1}\underset{A_{p}\cap A_{q}=A_{i}}{\sum }m_{1}\left(\;A_{p}\;\right)m_{2}\left(\;A_{q}\;\right) \end{array}$$

where

(3) $$\begin{array}{c} K=\underset{A_{p}\cap A_{q}=\emptyset }{\sum }m_{1}\left(\;A_{p}\;\right)m_{2}\left(\;A_{q}\;\right) \end{array}$$

K is the conflict between the two sources.

Dempster-Shafer uses ‘belief' rather than probability. Belief function is used to represent the uncertainty of the hypothesis.

To evaluate the uncertainty of the hypothesis, two functions can be calculated from a mass distribution: the belief function (Bel) and the plausibility function (Pls). Belief and plausibility functions can be considered as lower and upper estimations of probabilities.

(4) BelAi=∑Aj⊆AimAjPlsAi=∑Aj∩Ai=∅mAj

Bel(A) = 0 represents lack of evidence about A.

## 3. Results and Discussion

### 3.1. Modelling Uncertainty of AuxRE Detection

The objective of our study is detection of AuxRE. We applied a data fusion approach which consists of a combination of predictions coming from two techniques commonly used in pattern finding: overrepresented motifs and linear discriminant analysis. The idea is to extract, for each method, some features (parameters) and combine these parameters using the Dempster-Shafer (DS) rule, called orthogonal sum. We have applied our model to the* Arabidopsis thaliana* genome. The* Arabidopsis* genome sequence was obtained from TAIR [68].

Two hypotheses are involved: “this motif is an AuxRE”: “this motif is not an AuxRE” (i.e., not a motif or a motif other than AuxRE). In terms of the Dempster-Shafer evidence theory, we are in the case where the frame of discernment is constructed of two single hypotheses H1 and H2 and one composite hypothesis H3= H1 U H2 (union of H1 and H2). H3 represents in fact the ignorance.

**Figure 1:**
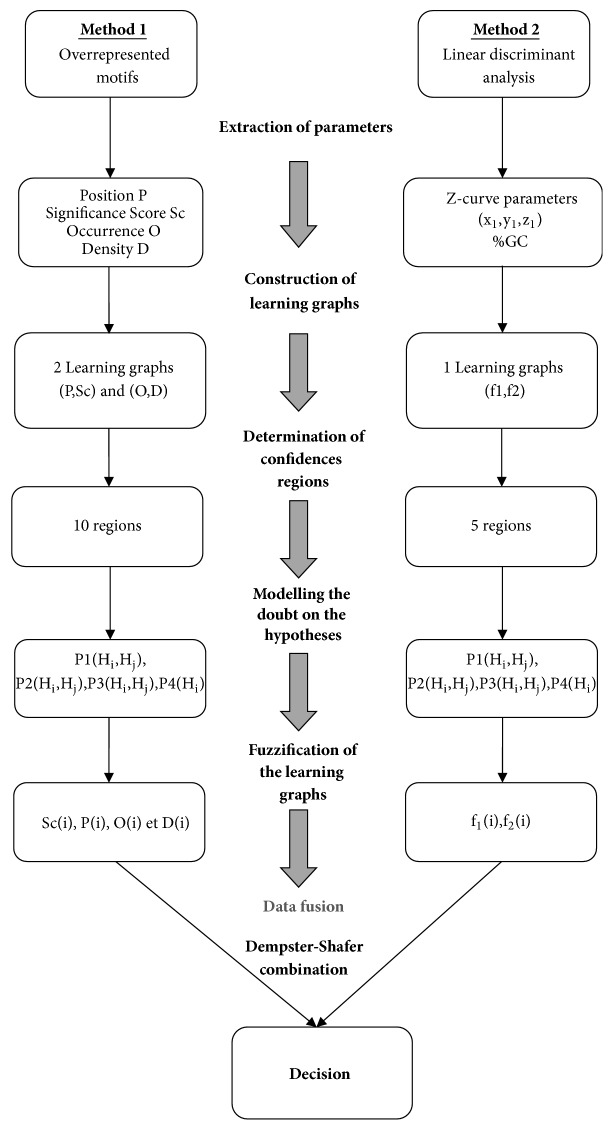
Modelling approach.

The modelling process is proceeding with six major steps (Figure 1):

- Step 1: extraction of parameters
- Step 2: construction of learning graphs
- Step 3: determination of confidence regions
- Step 4: modelling the doubt on the hypotheses
- Step 5: fuzzification of the learning graphs
- Step 6: data fusion methodology

#### 3.1.1. Extraction of Parameters

From the first method (detection of overrepresented motifs), we have prepared four parameters which are position P, significance score Sc, occurrence O, and density D. The position was located from the ATG. Significance score obtained from Weeder algorithm [54]. The occurrence represents the total number of a validated motif sequence in the whole genome of* Arabidopsis thaliana*. We have considered the density as the rate of a validated AuxRE motif sequence in promoter (-1000 bp) of response gene of Auxin. To prepare density, we have extracted the 2-fold Auxin-response gene from the microarray data.

(5) $$\begin{array}{c} D=\frac{Number\;\;of\;\;a\;\;validated\;\;motif\;\;sequence\;\;in\;\;the\;\;promoters\;\;of\;\;2\;\;folds\;\;Auxin\;\;resonse\;\;gene}{Total\;\;number\;\;of\;\;a\;\;validated\;\;motif\;\;sequence\;\;in\;\;all\;\;the\;\;promoters\;\;of\;\;arabidosis\;\;genes} \end{array}$$

We used the Z-curve parameters [69] and the GC% as potentially discriminative parameters and we performed a linear discriminant analysis. The Z-curve is a unique three-dimensional curve representation of a DNA sequence. We used three Z-curve parameters which are

(6) x1=a1+g1−c1+t1y1=a1+c1−g1+t1z1=a1+t1−g1+c1

#### 3.1.2. Construction of Learning Graphs

In the following sections, two methods will be presented that use the available data on a positive and a negative training set to construct a discriminative prediction model. A training set of 64 experimentally proven hormone response elements were collected from published data.

**Figure 2:**
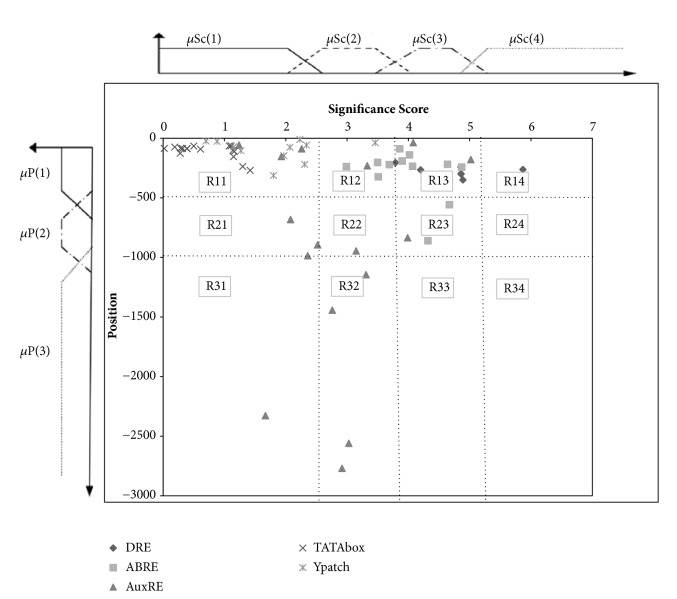
Learning graph 1: distribution of different type of motifs in significance score/position feature space.

**Figure 3:**
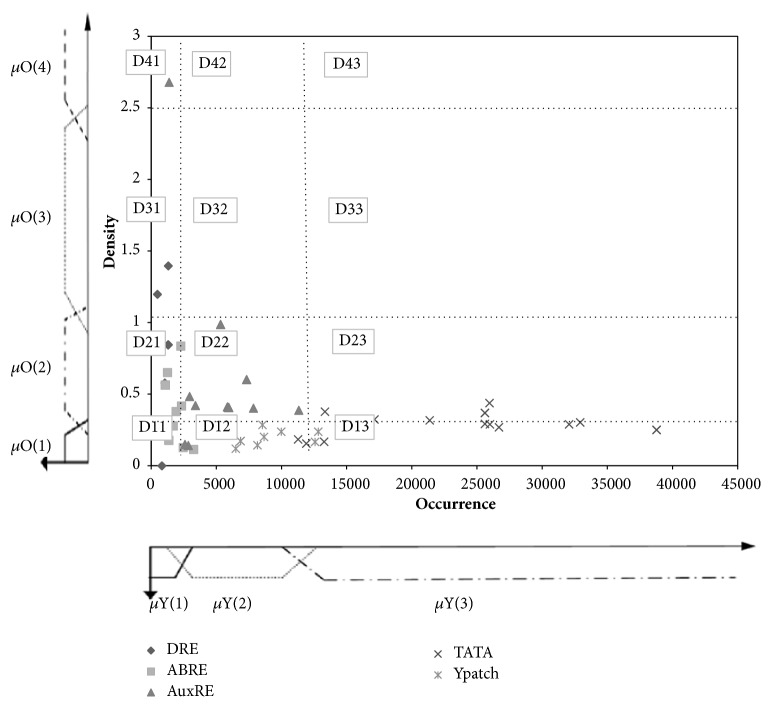
Learning graph 2: distribution of different type of motifs in occurrence/density feature space.

*Method 1: Overrepresented Motifs.* First, the validated motifs are studied in feature spaces which make the interpretation of the link between the selected features (P, SC, O, and D) and the type of motifs straightforward. We chose to study separately knowledge from position P and significance score Sc and those provided by occurrence and density in order to separate as much as possible AuxRE from other types of cis-regulatory elements. Two learning graphs have been created (Figures 2 and 3).  Figure 2 represents the distribution of validated motifs according to their parameters position P and significance score Sc. We distinguish, at the bottom of the graph, a region containing only AuxRE; the other part of the graph corresponds to an area of uncertainty which contains all types of motifs. This figure shows that only AuxREs are located relatively far from the translational start site (start codon). However, it is not a discriminative parameter, as many AuxREs were found in -500 bp upstream regions. Therefore, we have decided to study two other parameters (occurrence and density) in order to improve the classification and try to differentiate AuxREs, especially those found in the mixed region shown in Figure 2.

Figure 3 illustrates the classification of training cis-elements based on two parameters: the occurrence of the patterns in the -1000 bp upstream regions and the density.

**Figure 4:**
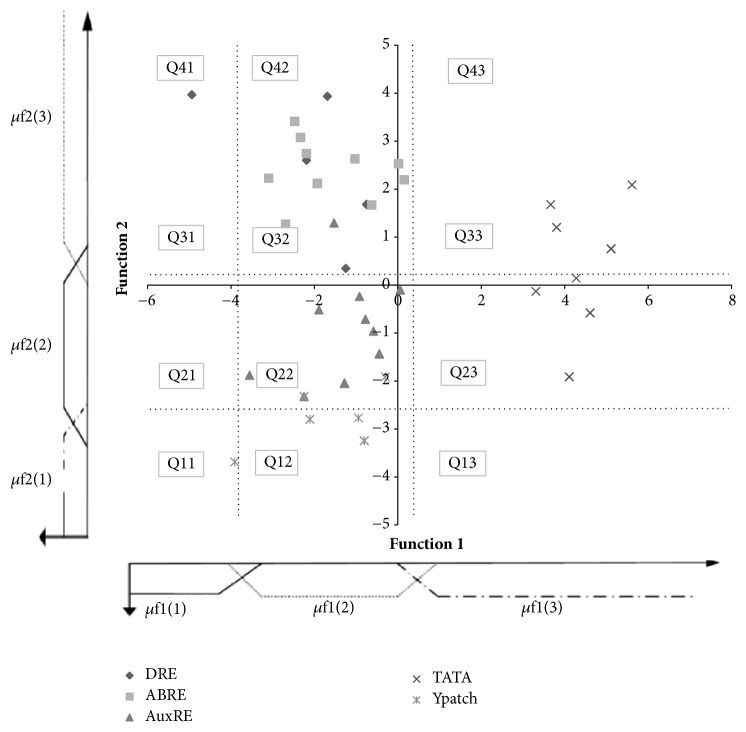
Learning graph 3: distribution of different type of motifs in f1/f2 feature space.

*Method 2: Linear Discriminant Analysis*. For the linear discriminant analysis, we have used the Z-curve parameter and the % GC.  Figure 4 shows the first two discriminant functions which allow a good discrimination of AuXRE from other motifs except Ypatch. The first discriminant function explains 59.6% of variability and has the highest correlation with GC% (-0.88) and Z1 (0.85) while the second function (32% of variability) is correlated to X1 (0.75).

#### 3.1.3. Confidence Regions

All the previous graphs do not allow a clear discrimination of AuxRE from other motifs. Each graph can be subdivided in several ways into different regions that will be enriched in one or few motifs. Here, we have chosen to partition the graph into five confidence regions shown in the Figures 1, 2, and 3 based on the percentage of AuxRE that belong to this region. The graph partition is given in Figures 1, 2, and 3 and Tables 2, 3, and 4.

#### 3.1.4. Modelling the Doubt on the Hypotheses

In order to make the graph partition an automatic process we attributed a confidence level to any unknown detected motif that would be located on the graph.

For that purpose, we define a gradual doubt through a set of four propositions:

- P1(Hi,Hj): total ignorance
- P2(Hi,Hj): low preference for the Hi hypothesis but high doubt between Hi and Hj
- P3(Hi,Hj): strong preference for the Hi hypothesis but low doubt between Hi and Hj
- P4(Hi): total confidence in the Hi hypothesis, no doubt

Next, these propositions are translated in terms of masses as detailed in Table 5. The preference level for a hypothesis from P1 to P4 is gradually represented by a mass value, respectively, equal to 0, 0.33, 0.67, and 1 [66]. Likewise, the gradual doubt between hypotheses is modelled by a mass value. In case of total doubt, the mass value affected equals 0. On the other hand, the mass value assigned to the total confidence is equal to 1.

Finally, a proposition is assigned to each region from the previous analyses on percentages of AuxRE and other motifs in each region. The link between the percentages and the related proposition are presented in Tables 2, 3, and 4.

#### 3.1.5. Fuzzification of the Learning Graphs

In the previous section we used discrete representation to define regions, which is not very objective because it can allocate confidence significantly different, for two near motifs from either side of boundaries. Moreover, the boundaries between regions are not well defined, and the transition from one region of the graph to another is not abrupt but a smooth one. Thus, In order to have a fuzzy, gradual continuous transition, we introduce the fuzzy logic theory. Therefore, we define fuzzy sets for each measured feature to predict its membership degrees to different possible feature families. For the parameter significance score four sets were defined (small, average, high, and very high). For the parameter position, three sets were described (core, proximal, and distal). For the parameters occurrence and density, three sets were defined (small, average, and high) for each of them.

#### 3.1.6. Data Fusion Methodology

The process of data fusion consists of fusing a number of learning graphs based on the definition of the so-called masses.

For each detected motifs, three masses are calculated, corresponding to the three learning graphs. They are given, respectively, by

(7) mO∈S/Sc&P=∑i=1,j=1i=4,j=3μScix∗μpjy∗mRijO∈S/Sc&PmO∈S/O&M=∑i=1,j=1i=3,j=3μOix∗μMjy∗mRijO∈S/O&MmO∈S/f1&f2=∑i=1,j=1i=3,j=3μf1ix∗μf2jy∗mRijO∈S/f1&f2

where S represents any subset of the hypotheses and *m*(*O* ∈ *S*/*Sc*&*P*), *m*_*R*_*ij*__(*O* ∈ *S*/*O*&*M*), *m*_*R*_*ij*__(*O* ∈ *S*/*f*1&*f*2) designate the mass corresponding to the region Rij of, respectively, the significance score/position graph, occurrence/density graph, and f1/f2 graph.

First, we have to fuse the two masses of method 1; this masse *m*_1_(*O* ∈ *S*) is obtained by combination of the two masses from the two feature spaces of method 1 through using the orthogonal sum of Dempster:

(8) m1O∈S=mRijO∈S/Sc&P⊕mRijO∈S/O&M

The final mass function is then calculated by fusing the two masses *m*_1_(*O* ∈ *S*) and *m*_*R*_*ij*__(*O* ∈ *S*/*f*1&*f*2); the orthogonal sum of Dempster is

(9) $$\begin{array}{c} m_{fusion}\left(\;O\in S\;\right)=m_{1}\left(\;O\in S\;\right)⊕m_{R_{ij}}\left(\;O\in S/f1\&f2\;\right) \end{array}$$

### 3.2. Scan of the Auxin Responsive DRN Promoter

**Figure 5:**
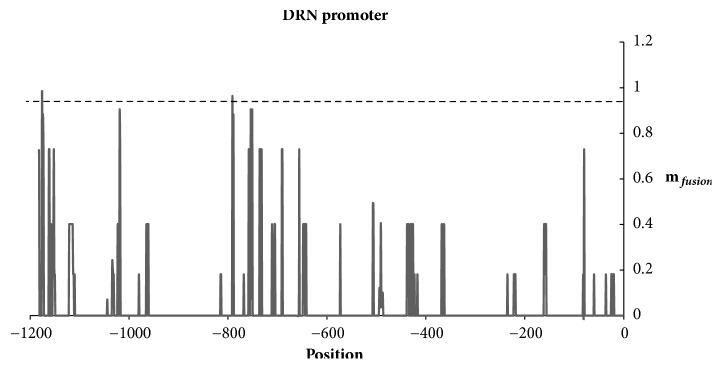
Scanning of the DRN promoter by the data fusion method.

DORNRÖSCHEN (DRN) promoter is one of the most studied Auxin responsive promoters which have an essential role in Auxin transport and perception in the* Arabidopsis* embryogenesis [70]. Two AuxREs that are not used in training have been experimentally identified in this promoter. To verify the reliability of the prediction, we tested our method to the DRN promoter. At a threshold of 0.9, the scanning of the DRN promoter by the model has detected the two validated AuxREs and at the same time we have not detected a false positive. Among 1200 motifs, we considered the two proven AuxREs as a true positive and the others as false positives (Figure 5).

### 3.3. Comparison between Method 1, Method 2, and Fusion

**Figure 6:**
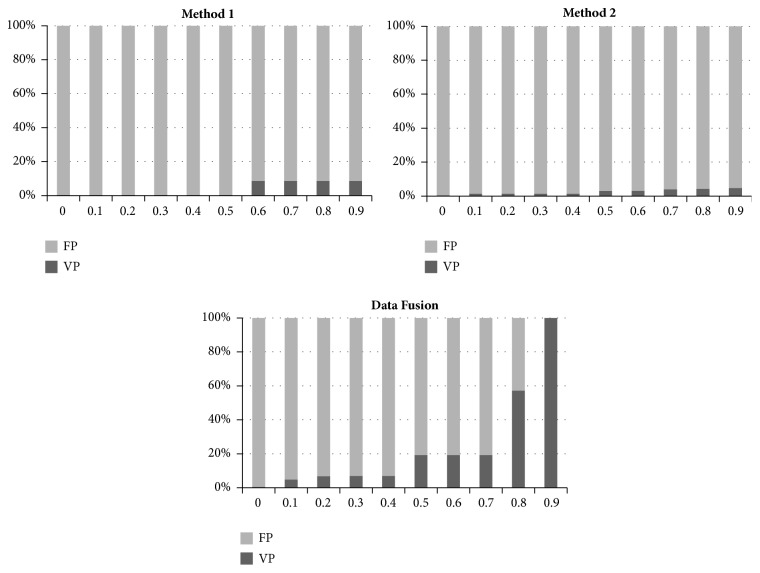
Evolution of the positive and false detection as a function of credibility obtained before and after data fusion.

**Figure 7:**
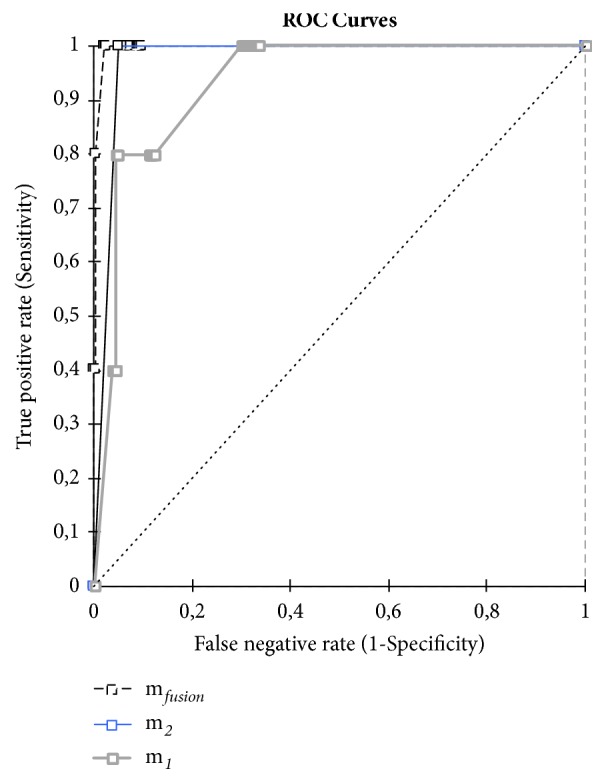
ROC curves before and after data fusion (scan of DRN promoter).

In order to study the influence of the data fusion by Dempster-Shafer combination, we have presented in Figure 6 the ration between true and false positive before and after combination. Figure 6 shows that, based on method 1 and method 2 separately, we have a large number of false positives. Their percentage exceeds 90% in both cases. After combination, it appears that the number of false positive significantly decreases to the point of cancelled when the credibility value equals 0.9. The reliability of detection is improved by data fusion. In parallel, the comparing of Tree ROC curves as shown in Figure 7 confirms the higher predictive reliability of the model after fusion compared with that based on only one method, when we scan DRN promoter.

### 3.4. Scan of DRN Promoter by Other Methods

To evaluate our method, we have scanned the DRN promoter by previous tools: Consensus [71], MEME [20], Gibbs Sampler [25], MDScan [72], and Weeder [54]. On the analysis platform MELINA II [73], the result indicates that the four motifs finding tools do not detect any AuxRE. These basic tools are unable to detect specific hormone responsive elements, but they detect cis-elements in general. We have also compared our model to the PWM method. PWM detects the two AuxREs but in return it produces a high frequency of false positive predictions. In fact, four false positives have been detected at a threshold equal to 0.9. For example, PWM detects the motif TTGTCAAA as an AuxRE with a score equal to 0.93 because this motif sequence is similar to the AuxRE sequence and, on the other hand, the PWM is based only on the composition. Conversely, this motif was not detected with our method since the prediction depends on several parameters. Likewise, the Plant Promoter Database (PPDB) has not detected these two validated AuxRE present in the DRN promoter. In this database, cis-regulatory elements are identified by the Local Distribution of Short Sequences (LDSS) and a prediction method based on microarray data methods (RARf-based approach)[74].

### 3.5. Scan of RD29B Promoter

The promoter of RD29B gene contains no AuxRE according to the literature. Several studies have shown the presence of other types of cis-regulatory elements such as ABA and DRE. The scan of this promoter by our model did not detect any false positives.

### 3.6. Validation of the Results

Because of the limited number of confirmed Auxin responsive elements, there is not enough data to divide it into training and validation sets. So, we have performed the Gold Standard [75] test to evaluate our model. A library of random DNA sequences (100 sequences) was generated using Unipro UGENE software version 1.26.1. (http://ugene.unipro.ru/) [76]. A set of 14 AuxRE was prepared. In each randomly generated DNA sequence only one AuxRE from preparing set was inserted at a random position using SeqKit toolkit [77]. A TSV file which contains a list of the sequences of inserted AuxRE and their positions of insertion was generated using csvtk (https://github.com/shenwei356/csvtk).

**Figure 8:**
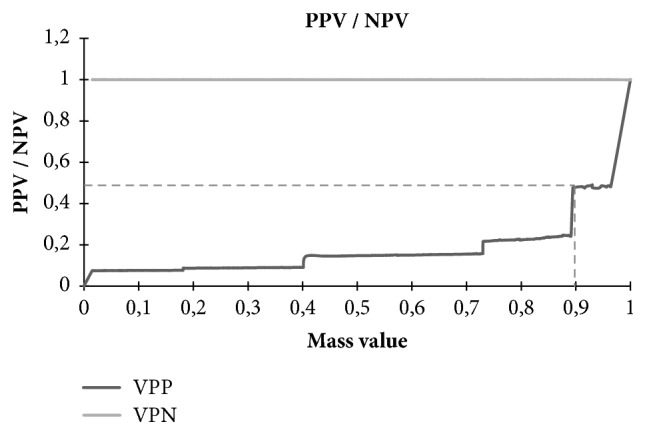
Variation of Positive Predictive Value (PPV).

In the next step, to further investigate the prediction performance and to choose the optimum cutoff, we applied our prediction method and we look at the variation of Positive Predictive Value (PPV). The results showed that we achieve maximal PPV for a cutoff value of 0.9 (Figure 8).

For an overall evaluation we have chosen to present the performance of our approach in comparison with other methods. The chosen methods are the five individual TFBS prediction tools evaluated by Jayaram et al. [78].

We do this by first summing true/false positives and negatives, and then statistical parameters were calculated in order to illustrate the best predictive approach.  Table 6 presented the obtained results. Our method is based on the joint using of Dempster-Shafer (DS) evidence theory and fuzzy sets and has the high degree of sensitivity (Sn) and Positive Predictive Value (PPV) with a value of 79 and 48.17, respectively, compared to the best previous methods. Even the Youden index (YI) and the Χ2 test parameters generated higher value than the other reference tools. Moreover, Table 6 shows that our approach (Data fusion) followed by the Clover computer program implemented by Frith et al. [42] are the best performing transcription factor binding sites (TFBS) prediction tools for individual sites. On the other side, Table 6 shows that the Find Individual Motif Occurrences (FIMO) method described by Grant et al. [44] has the worst sensitivity (Sn=22) on all the six presented tools. Besides, position specific scoring matrices (PoSSuMsearch) developed by Beckstette et al. [45] and FIMO tool have lower Positive Predictive Value (PPV) than the other previous methods, with a value of 40.74 and 42.31, respectively.

Our method strikes a good balance between sensitivity and PPV.

## 4. Conclusion

In this study, we applied a data fusion approach for the prediction of Auxin-response elements. Our method is based on the combined use of Dempster-Shafer (DS) evidence theory and fuzzy theory. We have tested our model to the DRN promoter and we have compared the prediction to previous tools. The results show that false positives are significantly decreased.

## Figures and Tables

**Table 1:** Datasets.

**Cis-regulatory elements**	**abbreviations**	**Numbers**	**References**
**Auxin**-response element	AuxRE	16	[27–37]
**ABA** response element	ABRE	12	[38–40]
**TATA** Box	TATA Box	16	[41]
**Y**patch	Ypatch	11	[41]
**drought**-responsive element	DRE	9	[38, 39]

**Table 2:** Proportion of false positive and true positive in the regions of the significance score/position feature space and associated propositions.

**Region R**_**ij**_	%** AuxRE**	%** non AuxRE**	**Proposition**
Z1: R11	7	93	P4(H2)
Z2: R12	25	75	P2(H2)
Z3: R13	0	100	P4(H2)
Z4: R14, R24, R34	0	100	P4(H2)
Z5: R23	80	20	P3(H1)
Z6: R21, R31, R32, R33, R22	100	0	P4(H1)

**Table 3:** Proportion of false positive and true positive in the regions of the occurrence/density feature space and associated propositions.

**Region Dij**	%** AuxRE**	%** non AuxRE**	**Proposition**
Z7: D11	43	57	P1
Z8: D12	13	87	P3(H2)
Z9: D22	91	9	P3(H1)
Z10: D41	100	0	P4(H1)
Z11: D21, D31	0	100	P4(H2)
Z12: D13, D23, D32, D33, D42, D43,	0	100	P4(H2)

**Table 4:** Proportion of false positive and true positive in the regions of the f1/f2 feature space and associated propositions.

**Region Q**_**ij**_	%** AuxRE**	%** non AuxRE**	**Proposition**
Z11:Q11, Q21, R12	0	100	P4(H2)
Z12: Q22	70	30	P3(H1)
Z13: Q13, Q23, Q33	0	100	P4(H2)
Z14: Q31, Q32	10	90	P3(H2)
Z15: Q41, Q42, Q43	0	100	P4(H2)

**Table 5:** Association of propositions with mass values.

**Proposition**	**m(H1) (AuxRE)**	**m(H2) (Pas AuxRE)**	**m(H1 U H2) (ignorance)**
P1(H1,H2)	0	0	1
P2(H1,H2)	0,33	0	0,67
P3(H1,H2)	0,67	0	0,33
P4(H1)	1	0	0
P2(H2,H1)	0	0,33	0,67
P3(H2,H1)	0	0,67	0,33
P4(H2)	0	1	0

**Table 6:** Comparison between our method and the other published methods.

	*Sn*	*Sp*	*PPV*	*NPV*	*FPR*	*FNR*	*YI*	*QCY*	*Χ*_*2*_	*Ref*
**Data fusion**	**79**	99.91	**48.17**	**99.98**	51.83	0.02	**0.79**	1	**36490.3**	
**Clover**	**69**	99.92	**47.9**	**99.97**	52.08	0.03	**0.69**	1	31702.8	[42]
Matrix-Scan	51	99.94	46.36	99.95	53.64	0.05	0.51	1	22661.6	[43]
Patser	63.64	99.92	43.45	99.96	56.55	0.04	0.64	1	27285.9	[43]
FIMO	22	99.97	42.31	99.92	57.69	0.08	0.22	1	8911.9	[44]
PoSSuMsearch	56.41	83.84	40.74	90.71	59.26	9.29	0.4	0.74	30	[45]

Average sensitivities (*Sn*). Specificity (*Sp*). Positive Predictive Value (*PPV*). Negative Predictive Value (*NPV*). False Positive Rate (*FPR*). False Negative Rate (*FNR*). Youden index (*YI*). Q coefficient of Yule (*QCY*) and *Χ*_2_ test value (*Χ*_2_). The best-performing tools. Data fusion and Clover are highlighted in bold.

## Data Availability

All the data used in this manuscript are included within the article and will be freely accessible upon its publication in BioMed Research International.
